# Histone Deacetylases in Herpesvirus Replication and Virus-Stimulated Host Defense

**DOI:** 10.3390/v5071607

**Published:** 2013-06-27

**Authors:** Amanda J. Guise, Hanna G. Budayeva, Benjamin A. Diner, Ileana M. Cristea

**Affiliations:** Department of Molecular Biology, Princeton University, Washington Road, Princeton, NJ 08544, USA; E-Mails: aguise@princeton.edu (A.J.G.); budayeva@princeton.edu (H.G.B.); bdiner@princeton.edu (B.A.D.)

**Keywords:** herpesvirus, HDAC, IFI16, acetylation, NF-κB, p53, proteomics, metabolomics, chemoproteomics

## Abstract

Emerging evidence highlights a critical role for protein acetylation during herpesvirus infection. As prominent modulators of protein acetylation, histone deacetylases (HDACs) are essential transcriptional and epigenetic regulators. Not surprisingly, viruses have evolved a wide array of mechanisms to subvert HDAC functions. Here, we review the mechanisms underlying HDAC regulation during herpesvirus infection. We next discuss the roles of acetylation in host defense against herpesvirus infection. Finally, we provide a perspective on the contribution of current mass spectrometry-based “omic” technologies to infectious disease research, offering a systems biology view of infection.

## 1. Introduction

Protein acetylation has recently emerged as a critical regulatory factor during herpesvirus infection. Recent studies have linked chromatin remodeling and acetylation/deacetylation events to the regulation of promoter activity and maintenance of viral latency during herpesvirus infection. These studies examined herpes simplex virus 1 (HSV-1), human cytomegalovirus (HCMV), Kaposi’s sarcoma-associated herpesvirus (KSHV) and Epstein-Barr virus (EBV) [1,2,3,4], thereby pointing to similarities among alpha-, beta- and gamma-herpesvirus infections. During latent infection, the HSV-1 genome is assembled into an ordered nucleosome-associated structure; however, during active lytic infection, the structure of viral chromatin exists in a more disordered state [5,6]. In lytic stages of infection, histones associate with herpesvirus promoter regions, and chromatin adopts a conformation consistent with more actively transcribed host genomic regions [6]. Moreover, it is well established that the post-translational modification of herpesvirus-associated histone tails is important for the regulation of viral gene transcription [7,8,9,10,11]. 

Given the significance of acetylation during viral infection and its requirement for essential host functions, knowledge of the regulation of the enzymes controlling protein acetylation is critical for understanding viral pathogenicity and host defense. This position is further emphasized by the recent finding that acetylation is a more prominent post-translational modification than previously thought, with several thousand host protein acetylations identified to date [12,13]. Among the enzymes involved in regulating protein acetylation are the human histone deacetylases (HDACs), which remove acetylations from their substrates [14]. Along with the numerous histone acetyltransferases (HATs) responsible for lysine acetylation, HDACs control the activity of substrates by acting as components of diverse multi-protein complexes with co-repressor functions (reviewed in [15,16]). HDACs themselves are a conserved family of proteins that evolutionarily predate histones, indicating that their interactions with non-histone proteins are integral to their cellular functions [17]. Given the array of human deacetylases, which encompasses eleven Zn^2+^-dependent HDACs and seven NAD^+^-dependent sirtuins (SIRTs), as well as their ability to participate in multiple protein complexes, viral interactions with members of this enzymatic family are likely to be protein-specific and individually regulated. HDACs have been linked to viral replication and pathogenesis during infection with a variety of human pathogens, including herpesviruses, hepatitis B and C, HIV-1 and HPV (reviewed in [18]). It is therefore not surprising that, during co-evolution with their hosts, viruses have gathered finely tuned mechanisms for targeting HDACs to either appropriate or inhibit their enzymatic activities. These observations highlight the importance of understanding the mechanisms of HDAC regulation during viral infection.

In this review, we summarize the mechanisms employed by herpesviruses to perturb the functions of this important family of host transcriptional regulators, the histone deacetylases. Next, we discuss the roles of acetylation in regulating host defense mechanisms against herpesvirus infection. Finally, we provide a perspective on the promise of emerging “omic” technologies for gaining a systems biology view of infection and an in-depth understanding of virus-induced changes within cellular pathways.

## 2. Viral Control of HDAC Complexes

**Figure 1:**
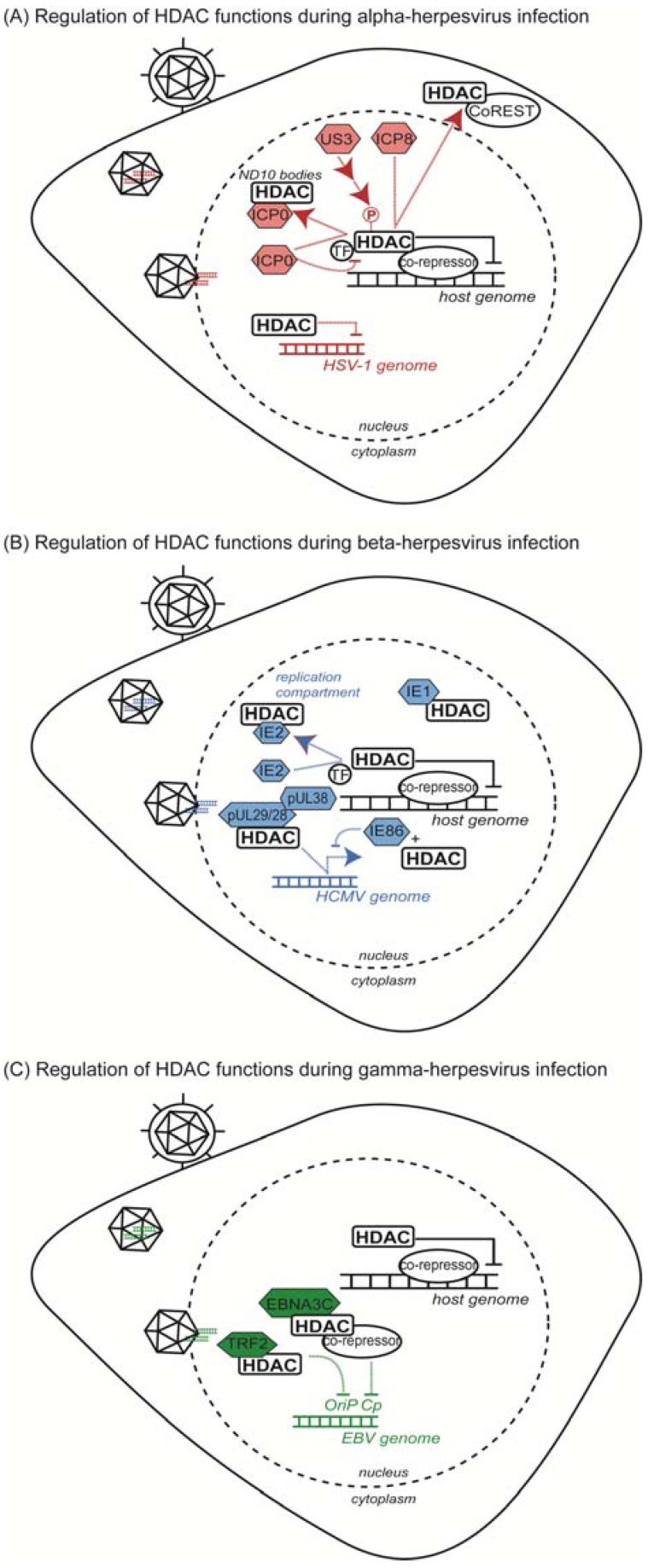
Summary of HDAC functions during herpesvirus infection. (**A**) Response to alpha-herpesvirus infection (HSV-1); (**B**) response to beta-herpesvirus infection (HCMV); (**C**) response to gamma-herpesvirus infection (EBV and KSHV).

Histone deacetylases are critical chromatin-associated transcriptional regulators responsible for the removal of lysine acetylations within targeted genomic regions, thereby promoting compact chromatin organization and repression of transcription. HDACs perform their repressive functions as components of numerous multi-protein co-repressor complexes, including the nucleosome remodeling and deacetylase (NuRD), co-repressor of RE1 silencing transcription factor (CoREST), mSin3A co-repressor, nuclear co-repressor (NCoR) and mitotic deacetylase (MIDAC) complexes [19,20,21,22,23,24,25,26]. The diversity of HDAC-containing complexes was clearly demonstrated by a recent proteomics-based study that profiled the interactions of all eleven human HDACs and assessed the relative stabilities of interactions within protein complexes [27]. This study demonstrated that HDACs can be part of numerous pre-assembled functional complexes that can associate with transcriptional factors for effective regulation of downstream gene expression. Moreover, these studies provided evidence that HDACs play roles not only in chromatin remodeling and transcriptional regulation, but also in diverse cellular processes, including cell cycle progression and RNA processing. Therefore, these results offer a valuable platform for examining changes in HDAC interactions during herpesvirus infection. The modulation of host protein interactions in response to infection provides both a mechanism for virus-mediated control of host protein functions for the benefit of viral replication, while also serving as a signal for the activation of host immune responses to counteract infection (summarized in Table 1 and Figure 1).

**Table 1:** Human histone deacetylase (HDAC) interactions with viral proteins during herpesvirus infection. HSV-1, herpes simplex virus 1; HCMV, human cytomegalovirus; EBV, Epstein-Barr virus; KSHV, Kaposi’s sarcoma-associated herpesvirus.

Enzyme	Virus	Interaction	Functional consequence	Ref.
**HDAC1**	HSV-1	ICP8	Redistribution of HDAC1/CoREST and LSD1 to cytoplasm	[28,29,30,31]
	HSV-1	ICP0	Disrupts CoREST association; localizes HDAC1/ICP0 to ND10 bodies	[28,32,33,34,35]
	HSV-1	US3	Upstream effector of HDAC1 phosphorylation	[32,36,37]
	HCMV	pUL29/28	Associates with HDAC1/HDAC2 and NuRD to promote expression of viral genes	[38,39]
	HCMV	pUL38	Associates with HDAC1/HDAC2 and NuRD complex members via pUL29/28 bridge	[38,39]
	HCMV	IE86	Co-expression promotes MIEP repression	[40]
	EBV	ENBA3C	Represses Cp promoter via association with co-repressor complexes (e.g., mSin3A and NCoR)	[41,42]
	EBV	TRF2	Deacetylation of OriP; promotes stability of latent genome	[43]
	KSHV	ORF50 promoter	Proposed to modulate promoter acetylation status and LANA acetylation	[2,44]
**HDAC2**	HSV-1	US3	Upstream effector of HDAC2 phosphorylation	[32,36,37]
	HCMV	pUL29/28	Associates with HDAC1/HDAC2 and NuRD to promote expression of viral genes	[38,39]
	HCMV	IE2	De-represses pUL54 promoter; promotes localization of HDAC2 to replications sites	[45]
	HCMV	pUL38	Associates with HDAC1/HDAC2 and NuRD complex members via pUL29/28 bridge	[38,39]
	EBV	ENBA3C	Represses Cp promoter via association with co-repressor complexes (e.g., mSin3A and NCoR)	[41,42]
	EBV	TRF2	Deacetylation of OriP; promotes stability of latent genome	[43]
**HDAC3**	HCMV	IE1	Increased acetylation at viral promoter	[46,47]
	HCMV	IE2	Increased acetylation at viral promoter	[46,47]
**HDAC4**	HSV-1	ICP0	Relieves MEF2-binding domain-mediated repression	[48]
**HDAC5**	HSV-1	ICP0	Relieves MEF2-binding domain-mediated repression	[48]
	KSHV	ORF50 promoter	Proposed to modulate promoter acetylation status and LANA acetylation	[2,44]
**HDAC7**	HSV-1	ICP0	Relieves MEF2-binding domain-mediated repression	[48]
	KSHV	ORF50 promoter	Proposed to modulate promoter acetylation status and LANA acetylation	[2,44]

### 2.1. Infection with the Alpha-Herpesvirus HSV-1 Promotes Misregulation of HDAC1/HDAC2-Containing Complexes

Several studies have started to uncover the means through which HDACs are recruited to viral protein-associated complexes and to viral genomes. As components of the CoREST complex, HDAC1 and HDAC2 act as repressors of host gene transcription within the nucleus. In the absence of infection, HDAC1 is observed within distinct nuclear structures in complex with CoREST and LSD1; however, upon HSV-1 infection, the HDAC1/CoREST/LSD1 complex is redistributed to compact structures that also contain the viral protein ICP8 (Figure 1A and Table 1) [28]. Resembling viral replication compartments, these structures suggest a function for this complex in active transcription of the viral genome [28]. As LSD1 is an established histone demethylase, this association can couple demethylase and deacetylase activities for coordinated regulation of histone post-translational modifications associated with the viral genome [28,29,30]. It is possible that the association of separate viral factors could trigger preferential association of specific accessory demethylases with the core CoREST complex. Upon infection, components of the HDAC1/CoREST/LSD1 complex are observed to translocate to the cytoplasm, indicating that this complex or its members may adopt new functions in response to viral infection (Figure 1A) [31]. Intriguingly, the redistribution of these complex members appears to be temporally regulated, with HDAC1/CoREST accumulating in the cytoplasm at four hours post-infection (hpi) and LSD1 changing localization at 8 hpi [28]. The HDAC1-CoREST interaction also contributes to a host defense mechanism against herpesviruses, which is discussed in Section 3.2 of this review.

Interestingly, the viral protein kinase U_S_3 can enhance expression of viral reporter genes, similar to the phenotype observed during HDAC inhibition, suggesting that a U_S_3-mediated kinase cascade may be an upstream regulator of HDAC activity [32]. Both HDAC1 and HDAC2 hyperphosphorylation during herpesvirus infection depends on the activity of U_S_3, yet HDAC phosphorylation appears to be an indirect effect of U_S_3 activity, suggesting the involvement of additional viral or host kinases (Figure 1A) [36,37]. In the absence of infection, phosphorylation is an established regulator of HDAC1 and HDAC2 activity and protein associations. Specifically, HDAC1 phosphorylation promotes binding to members of co-repressor complexes, including the Sin3a (RbAp48 and mSin3A), NuRD (RbAp48 and MTA2) and CoREST complexes [49]. Similarly, mSin3 and Mi2 (member of the NuRD complex) preferentially associate with phosphorylated HDAC2 [50]. The activity of viral kinases and perturbations in kinase signaling pathways could alter the phosphorylation landscape of these proteins, ultimately contributing to aberrant regulation of HDACs and rearrangement of HDAC complexes. 

Phosphorylations can directly mediate HDAC protein interactions; however, a second level of regulation results from phosphorylation-dependent changes in HDAC localization. Class IIa HDACs (HDAC4, -5, -7 and -9) shuttle between the nucleus and the cytoplasm through a mechanism that relies on site-specific phosphorylation of 14-3-3 binding sites [51,52,53,54]. Interestingly, the subcellular distribution of class IIa HDACs has also been observed to be dynamic during infection. HDAC4, -5 and -7 co-localize with ICP0 within the nucleus during HSV-1 infection (Figure 1A) [48]. ICP0 was shown to physically interact with these enzymes via the conserved *N*-terminal extensions shared by class IIa HDACs and absent in their class I counterparts [48]. ICP0 serves to relieve repression of MEF2 induced by the binding of HDAC *N*-terminal domains to MEF2. However, transcription mediated by the HDAC *C*-terminal deacetylation domain was not relieved by ICP0, consistent with continued co-localization of HDACs with SMRT, another member of the nuclear co-repressor complex [48]. bICP0 has also been reported to interact with HDAC1 during bovine herpesvirus 1 infection to modulate Mad-dependent transcription, suggesting that this virus-host protein interaction may represent a common point of virus-mediated control of host protein complex formation [33]. Therefore, the phosphorylation status of HDACs significantly contributes to the regulation of both protein interactions and localizations in the context of infection. Recent results have demonstrated that Aurora B-dependent phosphorylation of class IIa HDACs modulates cell cycle-dependent functions of these enzymes, indicating that HDAC phosphorylations are both spatially and temporally regulated [55]. The importance of temporally regulated phosphorylations during viral infection remains to be established.

Altogether, multiple viral factors, including ICP0, ICP8 and U_S_3, promote changes in the localization and functional roles of HDACs and co-repressor complexes during HSV-1 infection to promote viral reproduction. Further investigation of viral protein interactions with HDACs will contribute to development of a comprehensive understanding of the roles of individual HDAC family members during HSV-1 infection.

### 2.2. Beta-Herpesvirus HCMV Proteins Target Class I HDACs to Modulate Viral Gene Transcription

Modulation of HDAC complexes is also apparent during beta-herpesvirus infection. The HCMV proteins pUL29/28 and pUL38 interact with the HDAC1/HDAC2-containing NuRD complex in order to promote expression of immediate-early viral genes (Figure 1B and Table 1) [38,39] (Figure 1B). Immunoaffinity purification of pUL38 from cells infected with HCMV identified six components of the NuRD complex (Mi2β, MTA1, MTA2, HDAC1, HDAC2 and RbAp48/46 [38]), and the pUL38-NuRD interaction was shown to depend on the presence of the viral pUL29/28 protein [39]. Co-purification studies demonstrated that pUL29/28 binds to HDAC1 and MTA1 [39]. While pUL29/28 was also shown to interact with the Sin3A complex, this association seemed less stable than its interaction with NuRD, indicating preferential association of viral proteins to specific co-repressor complexes [39]. The pUL38/pUL29/28/NuRD complex forms early during infection and persists through 72 hpi, suggesting a role throughout multiple stages in the HCMV lifecycle [39]. While pUL29/28 and pUL38 exist in a complex together, these proteins also possess independent functions within the host cell. pUL38 interacts with TSC2, a component of the tuberous sclerosis tumor suppressor protein complex (TSC1/2), to maintain an active mTOR pathway during infection [38], whereas pUL29/28 was not shown to associate with TSC2 [39]. Moreover, pUL29/28 association with NuRD is independent of pUL38 and sufficient for transcriptional activation of the HCMV major immediate early promoter (MIEP). Treatment of cells infected with a pUL29/28-deficient virus with the HDAC inhibitor trichostatin A (TSA) rescued the decreased expression of immediate early genes induced by pUL29/28 depletion, demonstrating that this virus protein interaction with HDAC1/NuRD is important for stimulation of immediate early RNA production [39].

Similarly, HDAC inhibition was shown to relieve repression of other MIEP-dependent viral genes, as treatment with TSA rescued IE86-mediated autorepression [40]. IE86 interacts with HDAC1 and the histone methyltransferases G9a and Suvar(3–9)H1, likely contributing to changes in chromatin organization at the promoter region (Figure 1B) [40]. Co-expression of IE86 and HDAC1 enhanced repression of MIEP expression through both Rb-independent and -dependent mechanisms, indicating the potential involvement of multiple HDAC-containing complexes that differ in their association with Rb [40].

The immediate early proteins, IE1 and IE2, are also known to interact with HDACs to facilitate HCMV replication. IE1 and IE2 both interact with HDAC3, while IE2 was shown to also interact with HDAC2 (Figure 1B) [45,46]. Similar to the observations on IE86, HDAC inhibition rescued viral growth defects associated with IE1-deficient HCMV strains. Loss of IE1 triggers reduced histone H4 acetylation levels at the MIEP and the UL44 early promoter, indicating that HDAC activity at these regions is IE1-dependent [46]. IE2 interaction with HDAC3 is also thought to promote loss of deacetylation at viral promoter regions through a similar mechanism [46]. Intriguingly, overexpression of HDAC3 has been demonstrated to reduce activity from the MIEP and to limit HCMV infection [8]. As IE1 and IE2 are splice variants of the same mRNA sequence, domains common to both proteins could be important for association with structurally similar HDACs. Moreover, indirect association of immediate early proteins with bridge proteins could allow for interaction with multiple HDACs and HDAC-containing complexes [46,56,57]. Indeed, IE2 has been shown to associate with HDAC2 to de-repress expression from the viral polymerase (pUL54) promoter [45]. HDAC2 also exhibits altered localization during HCMV infection, co-localizing with IE2 at viral transcription and replication sites (Figure 1B); however, co-expression of IE2 alone was insufficient to induce HDAC2 redistribution, suggesting that additional viral factors contribute to its regulation during infection [45]. Interestingly, while the acetylation levels at the pUL54 promoter were observed to increase during the course of infection (from 6 hpi to 72 hpi), the acetylation at MIEP exhibited a different pattern—increasing at 24 hpi and diminishing at 48 hpi and 72 hpi. Therefore, HDAC-mediated regulation of viral promoter regions appears to be both promoter-specific and temporally regulated [45]. 

Careful regulation of gene expression is critical for progression through the viral lifecycle. The interaction of HCMV proteins with HDACs provides a mechanism by which viral gene programs can capitalize on the existence of an established host transcriptional regulatory system. HCMV proteins appear to display preference for individual HDAC-containing complexes, allowing for finely-tuned appropriation of host protein functions. Association of HDACs with viral promoters further allows modulation of the acetylation status of the viral genome, while simultaneously limiting the population of HDACs that would otherwise be available for interaction within typical host complexes.

### 2.3. The Gamma-Herpesviruses EBV and KSHV Regulate HDAC-Containing Co-Repressor Complexes through Protein Interactions and Phosphorylation-Dependent Signal Cascades

The gamma-herpesvirus EBV also modulates the activity of HDACs and HDAC complexes through the interaction of the viral Nuclear Antigen 3C (EBNA3C) protein with HDAC1 and HDAC2 (Figure 1C and Table 1) [41,42]. Careful regulation of viral gene expression is necessary for efficient replication, but also for maintenance of latency. The EBNA3C-HDAC interaction promotes the association of HDAC1 with the DNA binding protein CBF1/RPB-Jκ for both autorepression of EBNA3C and repression of Cp-responsive genes (Figure 1C) [41]. CBF1/RBP-Jκ is also reported to interact with HDAC1 and SMRT as part of a repressor complex [41,58]. As SMRT also associates with HDAC3 and class IIa HDACs, it is possible that multiple co-repressor complexes are recruited by EBNA3C to modulate complex-specific functions during infection [23,59]. Indeed, EBNA3C was subsequently shown to interact with HDAC1, HDAC2 and the co-repressor complexes mSin3A and NCoR as part of an EBNA3C-ProTα complex during EBV infection [42]. ProTα may serve as a bridge between EBNA3C and HDACs, as these co-repressor complexes were also demonstrated to associate with ProTα alone.

Viral gene transcription is further regulated by exploiting class IIa HDAC association with MEF2, which recruits HDACs 4, 5 and 7 to inhibit the viral BLZF1 promoter (Zp) [60,61]. Specifically, the de-phosphorylation of MEF2D has been shown to be important for regulating HDAC-mediated repression of Zp [61]. A consequence of this interaction is limited production of the immediate early viral protein BLZF1, which is a transcriptional activator important for exit from latency [60,61]. The temporal regulation of EBV gene expression is not only connected to HDAC transcriptional repressive functions (as in the case of MEF2), but also to deacetylation activity. HDAC1 and HDAC2 stably associate with the telomere repeat factor 2 (TRF2), which binds the EBV origin of plasmid replication (OriP) (Figure 1C) [43]. Based on the loss of HDAC/TRF2 binding to OriP during G1/S, along with a loss of deacetylation at OriP upon HDAC inhibition, this HDAC/TRF2 complex has been proposed to delay replication initiation from OriP in order to promote the stability of the latent EBV genome [43]. Thus, the temporal regulation of genome acetylation status and gene expression by HDACs is important for the preservation of latent stores of EBV within the host cell.

In addition to protein interactions, signaling pathways activated by viral infection affect HDAC activities, possibly through signal-dependent post-translational modification. The KSHV protein vGPCR has been reported to induce signaling pathways that converge on HDACs, among other targets [62]. vGPCR induces expression of the ORF50 promoter, which produces the protein RTA (replication and transcription activator) that, in itself, is sufficient for lytic reactivation of KSHV [62,63]. vGPCR activates PKC and PKD signaling pathways associated with regulation of ORF50 expression [62]. These kinases are well-established regulators of nuclear export of class IIa HDACs [51,64], indicating that HDAC phosphorylation during infection may be an important strategy for virus-mediated control of HDAC activity and complex formation. vGPCR signaling was reported to reduce the activity of deacetylases, as shown for both HDACs and sirtuins [62]. HDACs 1, 5 and 7 were shown to associate with the ORF50 promoter during KSHV latency, and the acetylation status of the latency-associated nuclear antigen (LANA) was dependent on HDAC activity [2,44]. The KSHV LANA protein can also interact with the HDAC co-repressor complex mSin3 during EBV infection [65]. Given the importance of temporal coordination of viral gene programs, cell cycle-dependent kinase cascades could provide an additional level of regulation of gene expression during infection. Interestingly, ORF50 is also capable of repressing p53 transcriptional activity through an interaction with the CREB binding protein, CBP [66,67]. As p53 activity is regulated by acetylation, it is tempting to speculate that p53-associated responses to viral infection are, in part, due to misregulation of deacetylase complexes [68,69].

Altogether, these studies have demonstrated that gamma-herpesviruses EBV and KSHV co-opt HDAC complexes to repress viral gene expression. These mechanisms likely allow for maintenance of the viral genome in a latent state until conditions are advantageous for viral replication. Viral infection further alters HDAC activity through activation of phosphorylation signaling cascades, thereby modulating the phosphorylation states and activities of individual HDAC enzymes.

## 3. Host Employment of HDACs and Acetylation in Defense against Herpesviruses

Comprehensive acetylome studies have revealed that histone and non-histone protein acetylation is comparable in frequency to phosphorylation [12,13]. One study alone identified over 3,500 acetylations across three human cell types using immunoaffinity isolation of acetylated substrates coupled with mass spectrometry analysis [13]. Interestingly, acetylations within several important effectors of innate immunity—RIG-I, IRAK4, OAS2, TRIM25 [13] and, most recently, IFI16 [70]—have been identified, suggesting that acetylation provides dynamic control of important mammalian innate immune functions. Indeed, increasing evidence suggests that HDACs are regulators of inflammatory response, immune signaling and myeloid differentiation [71,72,73,74]. 

### 3.1. IFI16 Acetylation and Host Detection of Viral DNA

**Figure 2:**
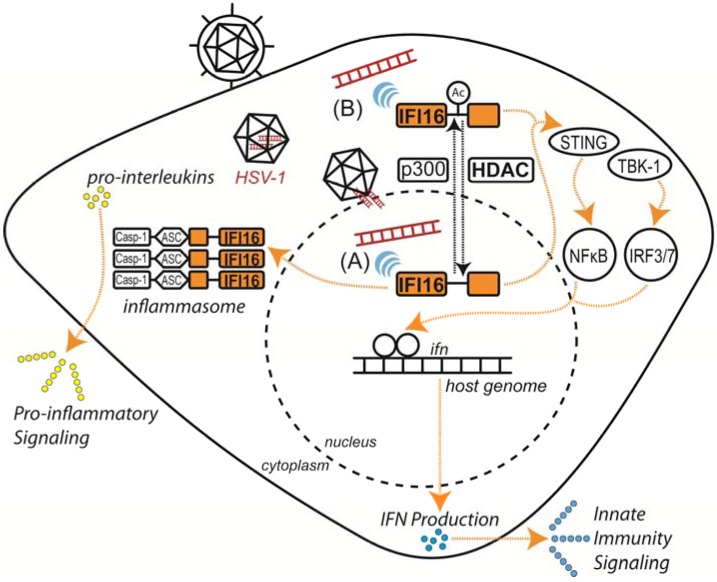
Host response to viral DNA is mediated by IFI16. Acetylation-dependent localization of IFI16 allows dual-compartment sensing of viral DNA, promoting pro-inflammatory response and innate immune signaling during herpesvirus infection. (**A**) IFI16 senses HSV-1 DNA within the nucleus. (**B**) IFI16 senses cytoplasmic DNA resulting from transient transfection or viral capsid degradation.

In mammals, induction of host innate immunity in response to viral infection begins with the detection of pathogen-associated molecular patterns (PAMPs) or danger-associated molecular patterns (DAMPs) by intracellular pattern recognition receptors (PRRs). Several classes of PRRs have been characterized, including AIM2-like receptors (ALRs), retinoic acid-inducible gene I-like receptors (RLRs), nucleotide oligomerization domain-like receptors (NLRs), Toll-like receptors (TLRs) and an assorted array of cytoplasmic DNA receptors [75]. These PRRs are predominantly localized to the cytosol, plasma membrane and endosome to sense and respond to a wide range of invading pathogens. Surprisingly, recent studies have extended the range of host immuno-surveillance to the nucleus, where the DNA sensor IFI16 selectively detects the double-stranded DNA genome of herpesviruses during infection [70] (Figure 2). 

As an ALR family member, IFI16 senses nuclear and cytoplasmic herpesvirus DNA, inducing pro-inflammatory and interferon responses via distinct pathways to limit viral replication and spread (Figure 2) [70,75,76,77,78,79,80]. In the cytoplasm, IFI16 associates with the signaling adapter protein STING (stimulator of interferon genes) upon binding of viral DNA, subsequently activating IRF3 and NF-κB transcription factors, which then translocate to the nucleus and induce robust interferon-β (IFN-β) expression [75,80]. However, DNA sensing is not restricted to the cytoplasm, as IFI16 has also been demonstrated to bind herpesvirus DNA in the nucleus during early stages of infection to induce IFN-β expression [70]. Intriguingly, following detection of viral DNA, IFI16 and the additional ALR AIM2 form oligomeric, multi-protein complexes known as inflammasomes [76,78,79,81,82]. In the cytoplasm, these inflammasomes recruit and activate caspase-1, which, in turn, processes the pro-inflammatory cytokines pro-IL-1β and pro-IL-18 to their secreted, biologically active forms. Until recently, the underlying mechanisms regulating these localization-dependent innate immune functions of IFI16 were not well-understood. Li *et al.* identified two acetylations within IFI16 that regulate its sub-cellular distribution [70]. Partly mediated by the HAT p300, acetylation of Lys99 and Lys128 within the nuclear localization signal (NLS) promotes cytoplasmic retention of IFI16, whereas HDAC activity promotes its nuclear import. Nuclear localization of IFI16 was shown to be essential for recognition of nuclear herpesviral DNA during infection [70,76,77], indicating that HDACs may play a critical role in IFI16-mediated DNA sensing. Thus, modification by acetylation provides a means for expanding the range of IFI16-mediated immuno-surveillance of double-stranded DNA viruses and may function as a toggle for additional localization-dependent functions (Figure 2). Observations of the multiple patterns of IFI16 behavior during viral infection suggest that this protein may have varied functions in immune response. While acetylation of IFI16 is critical for positioning this DNA sensor in the appropriate cellular compartment prior to infection, the roles of IFI16 acetylation and associated HDAC functions during infection require further investigation.

IFI16 is not the only PRR known to be acetylated. RIG-I, a sensor of viral double-stranded RNA that acts through the MAVS adapter protein to induce IFN signaling [83], is acetylated at Lys858 within its *C*-terminal repression domain [13]. While Lys858 acetylation has yet to be tested for modulation of RIG-I activity, acetylation of PRRs and HDAC modulation of PRR activities may be a more prominent regulatory feature of the innate immune system than currently appreciated.

Once PRRs bind their pathogen-derived ligands, innate immune signals are transmitted to neighboring cells via cytokine production and secretion. HDACs have indirectly been implicated in these processes. Animals treated with valproic acid (VPA), a broad-acting HDAC inhibitor, exhibit increased susceptibility to infection by bacterial and fungal pathogens, while being protected from septic shock [73]. In agreement with these observations, VPA-treated macrophages in the presence *E. coli* and *S. aureus* exhibit reduced phagocytosis and subsequent cytokine bursts [74]. Several reports have described attenuated expression of cytokines important in innate immunity signaling (e.g., IFN-β and IFN-γ), pro-inflammatory responses (e.g., TNF-α, IL-6, IL-1β and IL-18) and leukocyte invasion and activation (e.g., MCP-1, G-CSF and CXCL10) following treatment with the pan-HDAC inhibitor, TSA [73,74]. However, dampened cytokine expression does not necessarily correlate with repressive chromatin modifications, as histone H4 acetylation at *Tnf* and *IL6* promoters has been shown to increase following TSA treatment [73]. Thus, it is likely that a significant subset of the immunosuppressive, anti-inflammatory effects of HDAC inhibitors occur at a non-histone level. 

### 3.2. HDAC Association with PML/ND10 Bodies during Herpesvirus Infection

Associated with viral DNA after its nuclear deposition, PML bodies are composed of a set of interferon-inducible proteins that play important roles in host anti-viral defense during herpesvirus infection [84,85]. During HCMV infection, HDACs are recruited to the viral MIEP promoters by Daxx, a component of PML bodies, to repress viral gene expression at early stages of infection [47]. However, this intrinsic immune defense mechanism is counteracted by the activity of viral proteins. Specifically, the HCMV protein pp71 promotes degradation of Daxx, and the interaction of the immediate early viral proteins, IE1 and IE2, with HDAC3 relieves repression of viral transcription [46,47]. This mechanism of evading HDAC-mediated anti-viral response appears to be conserved across species, as the mouse CMV protein mIE1 is also reported to bind mHDAC2, which is recruited to ND10 structures via PML and Daxx [86]. Consistent with a model in which HDAC activity represses viral gene transcription through association with the HCMV MIEP, HDAC inhibition has been demonstrated to rescue IE gene expression [87]. During HSV-1 infection, disruption of ND10 structures is necessary for productive viral replication and is accomplished by ICP0 [88]. Moreover, ICP0 promotes the disruption of HDAC1-CoREST to enhance viral gene expression and replication [31,34]. An *N*-terminal domain of ICP0 shares homology with and binds to a *C*-terminal domain of CoREST immediately adjacent to the HDAC1 binding site [34]. Increased viral gene expression allows the growing population of ICP0 to outcompete HDAC1 in the binding of CoREST. Through this mechanism, ICP0 promotes inhibition of CoREST-associated gene repression by abolishing its interaction with HDAC1. Another viral protein, ICP8, also accumulates adjacent to ND10 structures, where it could function to preferentially bind DNA [28,35]. ICP0-mediated inhibition of HDAC activity is also proposed to facilitate the transition from the expression of viral α genes to β genes [32]. Thus, it is likely that viral protein associations with HDACs and HDAC-containing complexes are temporally regulated during the course of infection to coordinate gene expression for productive viral replication.

### 3.3. HDAC Non-Histone Substrates in Host Defense

Host response to viral infection is known to be regulated by multiple transcription factors, whose downstream targets include genes associated with apoptotic, immune and pro-inflammatory responses. Several of these transcription factors with critical roles in activation of anti-viral gene programs are known to be regulated by acetylation. For example, the interferon-α receptor (IFNAR) is acetylated at Lys399 following IFN-α binding, directly recruiting interferon regulatory factor 9 (IRF9), along with the signal transducer and activator of transcription 1 (STAT1) and STAT2 [89]. IRF9, STAT1 and STAT2 are all acetylated within their DNA-binding domains, and modification is thought to promote transcriptional activation at interferon-inducible promoters. Similarly, STAT3 dimerization is modulated by the p300-mediated acetylation at Lys685, which promotes nuclear accumulation of STAT3 and transcriptional activation following cytokine-induced signaling [90]. These results suggest that acetylation may be able to positively regulate innate immune signaling pathways, which is in apparent disagreement with global HDAC inhibition studies outlined above. However, recent evidence suggests that the acetylation of STAT1 serves to terminate IFN-α-induced signaling and that deacetylation of STAT1 by HDAC3 promotes its re-association with IFNAR [91]. Thus, HDAC-dependent reinstatement of the STAT1-IFNAR interaction may account for the discrepancy in models for acetylation-dependent regulation of innate immune signaling. As HDACs are effectors of innate immunity signaling, it is not surprising that multiple virus types would develop strategies to promote the disruption of HDAC activities during infection. 

Multiple subunits of NF-κB are also regulated by site-specific acetylation (reviewed in [92]). The functional consequences of post-translational modification of NF-κB include modulation of κB-DNA binding by NF-κB, transcriptional activation of NF-κB and association of NF-κB with its negative regulator, IκBα [93]. Specifically, Lys122 and Lys123 of the p65/RelA subunit of NF-κB were shown to be modified by p300/PKAF and HDAC3 [94]. Further analysis revealed that acetylation of NF-κB p65/RelA negatively regulates its binding to κB-DNA and facilitates its nuclear export. In contrast, NF-κB deacetylation has been linked to its activation. Interestingly, several studies have also demonstrated that deacetylation of p65/RelA by SIRT1 and SIRT2 limits NF-κB activity. This is achieved through either direct inhibition of NF-κB transcriptional activity or promotion of its association with IκBα, ultimately leading to diminished secretion of pro-inflammatory cytokines [95,96,97,98]. Thus, the association of NF-κB with different deacetylases may be an important determinant of its activity.

These NF-κB functions are carefully regulated during herpesvirus infection in order to redirect NF-κB activity for increased viral gene expression [99,100]. HSV-1 interferes with NF-κB association with the promoter of IκBα and, instead, recruits NF-κB to the ICP0 promoter to facilitate expression of this viral protein [99]. It is conceivable that the altered activity of NF-κB results, at least in part, from changes in its acetylation status, suggesting a possible mechanism by which NF-κB regulation can be exploited in the design of disease treatments. A novel cancer treatment method has recently been proposed, in which treatment of HSV-1 infected cells with TSA induces acetylation and nuclear accumulation of NF-κB, promoting virus production and decreasing the viability of tumor cells [101]. Emerging evidence suggests that NF-κB acetylation may be involved in host immune responses to multiple viruses [102], further highlighting the importance of understanding the regulation of NF-κB and the activity of the enzymes responsible for modulating its acetylation status.

Another critical non-histone HDAC substrate with roles in immune defense during herpesvirus infection is the tumor suppressor, p53. The pro-apoptotic functions of p53, as well as its ability to transactivate IFN-inducible genes during vesicular stomatitis virus (VSV) infection, are dependent on the acetylation state of Lys379, which is negatively regulated by SIRT1 [103]. Interestingly, during HCMV infection, the viral protein IE2 can downregulate p53 function through inhibition of the acetyltransferase p300/CBP [104]. Sequestration of p300/CBP and the transcription factor IRF-3 by ICP0 has also been observed during HSV-1 infection, resulting in attenuated immune response [105]. It has been proposed that HDAC-1, -2 and -3 can downregulate p53 transcriptional activity through deacetylation of residues within its *C*-terminus [106]. In view of these observations, further investigation of the regulatory functions of HDACs with respect to p53 activity during infection is necessary.

### 3.4. Roles for Additional Acetylation-Modulating Enzymes during Infection: SIRTs and HATs

Further highlighting the significance of protein acetylation during viral infection, recent studies have started to uncover roles for other acetylation-modulating enzymes. While not the focus of this review, it is worth mentioning that the NAD^+^-dependent deacetylases, sirtuins (SIRTs) and histone acetyltransferases (HATs) seem to be intimately linked to viral replication. Studies examining the functions of the seven mammalian sirtuins during the progression of viral infection are still limited. However, SIRT1 has been implicated in the progression of human immunodeficiency virus 1 (HIV-1) [102,107,108,109], human papillomavirus (HPV) [110] and vesicular stomatitis virus (VSV) [111] infection. SIRT1 has also been proposed to control the reactivation of latent varicella zoster virus (VZV) in neurons through the reduction of intracellular NAD^+^ levels [112]. Additionally, canine coronavirus (CCoV-II) infection has been shown to induce the expression of SIRT1, SIRT3 and SIRT4 [113], suggesting that the regulation of individual sirtuins is virus specific. Sirtuins possess diverse cellular functions, including modulation of gene expression, DNA repair and apoptosis. Such pathways are commonly exploited by herpesviruses in order to promote viral replication. Therefore, it is tempting to speculate that sirtuins, like the Zn^2+^-dependent HDACs, have roles in modulation of herpesvirus lifecycles through protein complex associations and regulation of substrate activities within essential pathways.

The modulation of HAT complexes and HAT activities during infection has proven to be an equally interesting subject for investigation. While this review focuses on the modulation of HDAC complexes during herpesvirus infection and the roles of HDACs in host defense mechanisms, it is worthwhile to briefly highlight several examples of roles for HATs during infection. During HSV-1 infection, the circadian HAT CLOCK has been shown to be recruited to ND10 bodies and viral replication compartments, where it associates with the viral proteins, ICP4, ICP27 and ICP22, and the transcription factor, TFIID [114,115]. CLOCK activity promotes the expression of *α*-genes [114] and, along with its regulator and substrate, BMAL1, remodels viral chromatin [115]. Like their deacetylase counterparts, HAT activities are also modulated by phosphorylation, and, indeed, phosphorylation of TIP60 by a conserved serine/threonine kinase encoded by HSV-1, HCMV, EBV and KSHV has been shown to activate TIP60 during herpesvirus infection and to induce DNA damage responses and chromatin remodeling [116]. TIP60 activity is proposed to have a negative effect on establishment of viral latency, instead promoting active lytic replication [116]. During bovine herpesvirus 1 infection, bICP0 interacts with p300 and may serve to inhibit p300-mediated interferon response to viral stress [117]. Altogether, the dynamic changes in the composition and activity of HAT complexes induced by interactions with viral proteins indicate that these enzymes, like HDACs, have critical roles during herpesvirus infection. 

In summary, multiple important regulators of host response to viral infection are dynamically regulated by acetylation and deacetylation, indicating that HDACs are essential upstream modulators of host anti-viral responses. Further analysis of HDAC substrates during viral infection will help define acetylation-dependent mechanisms involved in host response to pathogens, providing additional targets for the development of anti-viral therapeutics. 

## 4. Perspective: “Omic” Approaches in Characterizing HDAC Functions in Virus Infection

**Figure 3:**
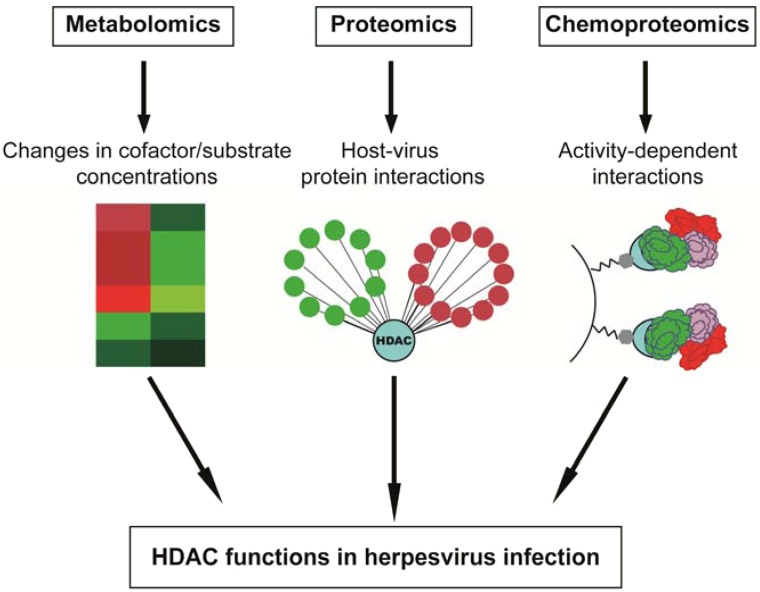
Assessing HDAC functions during infection through integrative omic approaches. A combination of multi-disciplinary approaches, including metabolomics, proteomics and chemoproteomics, can provide information for comprehensive characterization of the roles that HDACs play during herpesvirus infection.

As changes in the proteome, metabolome and lipidome of infected cells have started to be established as critical markers for infection, mass spectrometry-based techniques have become an invaluable resource for the field of infectious disease research. Integration of such “omic” methodologies promotes development of a systems biology view of viral infection, which offers a multi-dimensional understanding of the diverse virus-mediated changes in intracellular processes. The impact of such approaches is particularly apparent when considering the diverse functions of HDACs. Omic-based studies can provide insights into the molecular mechanisms underlying HDAC functions during viral infection, including elucidation of their histone and non-histone substrates, their participation in protein complexes and their impact on downstream transcriptional targets (Figure 3).

Developments in mass spectrometry and bioinformatics have provided powerful tools for quantifying protein levels and defining interactions and post-translational modifications. Quantitative mass spectrometry incorporating metabolic labeling has been successfully used to assess changes in protein expression levels during pseudorabies virus (PRV) infection, identifying alterations in expression patterns for proteins involved in intracellular transport, translation and host stress response [118]. Similarly, identification of dynamic host-virus protein interactions during infection has contributed significantly to the understanding of both virus-mediated effects on host cell functions and of host defense mechanisms in response to infection (e.g., [38,119,120,121,122,123,124,125,126]). The use of affinity purification coupled with mass spectrometry (AP-MS) has proven effective for both targeted and global protein interaction studies [127]. Furthermore, fluorescent affinity-tags have allowed integration of knowledge regarding protein interactions and localizations, thereby providing a spatial-temporal view of infection [119,128]. Construction of networks incorporating host-virus and virus-virus protein interactions readily illustrates the functional relationships among individual proteins and protein complexes during infection. Application of these methods has advanced the current understanding of virus-host protein interactions during HCMV [38,121,123] and PRV [126] infections. As discussed earlier in this review, proteomic approaches have helped establish the roles of HDACs during HCMV infection, as exemplified by the finding that the HDAC1-containing NuRD complex is recruited by viral proteins during infection [38,39].

On a larger scale, several recent AP-MS studies have examined global host-virus protein interactions, generating comprehensive interaction networks that further highlight the benefit of applying proteomic approaches to investigating viral infection [120,124,125,129]. Moreover, the resulting large-scale protein interaction networks are useful in the development and optimization of statistical methods for characterizing protein associations, including the SAINT algorithm for determining specificity of protein interactions [130]. Curated protein databases, including the HIV-1 Human Protein Interactions Database and VirusMINT, further integrate knowledge of viral protein interactions [131,132,133,134]. The expansion of these databases to include proteomic analyses for other viruses will be invaluable for future studies.

In addition to the identification of protein interactions, proteomic approaches allow for identification of post-translational modifications of both viral and host proteins. For example, a recent proteomic study of HSV-1-infected cells led to the identification of multiple phosphorylation and ubiquitination sites within viral proteins [135]. Future investigation and functional characterization of post-translational modifications, including HDAC-regulated acetylations, will expand the current understanding of mechanisms involved in the regulation of viral gene expression and replication. 

Alongside advances in AP-MS methodologies, chemoproteomics approaches have emerged as effective techniques for profiling changes in enzymatic activities and for studying activity-dependent protein interactions during infection. Activity-based protein profiling (ABPP) has been applied to the examination of ubiquitin proteases during herpesvirus infection [136]. Chemoproteomic approaches have also been used to resolve the composition of HDAC complexes and to assess the ability of small molecules to inhibit specific HDACs [26]. The development of probes allowing for site-directed capture of HDACs has enabled comprehensive characterization of HDAC substrates and of additional activity-dependent interactions [137,138]. Application of this method for the quantitative study of HDAC activity-dependent interactions during herpesvirus infection will further elucidate the molecular roles of HDACs during infection.

In conjunction with monitoring proteomic changes during infection, examination of alterations in metabolite stability can provide important insight into specific metabolic pathways employed during viral replication cycles. Developments in mass spectrometry-based techniques have allowed quantification of changes in cellular metabolism with increased accuracy [139,140]. Metabolomics studies following either HCMV or HSV-1 infection have revealed variations in host cell metabolic patterns that correlate with viral growth kinetics [139,141,142,143,144]. These studies demonstrated that herpesviruses can impact metabolic flux through individual pathways [144]. Specifically, HCMV infection induces glycolytic flux to fuel fatty acid biosynthesis, whereas HSV-1 preferentially stimulates production of components required for pyrimidine nucleotide biosynthesis. Interestingly, HSV-1 infection triggers a significant decrease in intracellular NAD^+^ levels that is not observed during HCMV infection, suggesting that NAD^+^-dependent deacetylases, SIRTs, may be differentially regulated in the presence of these viruses. Similarly, with developments in mass spectrometry-based lipidome studies [145], virus-induced changes in cellular lipids are becoming more apparent following infection with various types of viruses [146,147,148], including herpesviruses [149,150]. Overall, the identification of distinct cellular pathways and pathway components necessary for the replication and spread of individual viruses will allow for increased target specificity and selectivity in the design of anti-viral therapeutics [151].

As methodologies for proteomic, metabolomic and lipidomic studies, as well as necessary bioinformatics approaches, are constantly being adapted and improved, it is expected that these “omic” technologies will play an increasingly important role in infectious disease research. 
